# Azithromycin Attenuates *Pseudomonas*-Induced Lung Inflammation by Targeting Bacterial Proteins Secreted in the Cultured Medium

**DOI:** 10.3389/fimmu.2016.00499

**Published:** 2016-11-15

**Authors:** Teresinha Leal, Gabriella Bergamini, François Huaux, Nadtha Panin, Sabrina Noel, Barbara Dhooghe, Jeremy B. Haaf, Pierluigi Mauri, Sara Motta, Dario Di Silvestre, Paola Melotti, Claudio Sorio

**Affiliations:** ^1^Louvain Centre for Toxicology and Applied Pharmacology, Institut de Recherche Expérimentale et Clinique, Université catholique de Louvain, Brussels, Belgium; ^2^Cystic Fibrosis Translational Research Laboratory “D. Lissandrini”, Department of Medicine, Division of General Pathology, University of Verona, Verona, Italy; ^3^Cystic Fibrosis Center, Azienda Ospedaliera Universitaria Integrata di Verona, Verona, Italy; ^4^Institute for Biomedical Technologies (ITB-CNR), Segrate, Milan, Italy

**Keywords:** azithromycin, cystic fibrosis, cytokines, inflammation, proteomics, *Pseudomonas aeruginosa*

## Abstract

**Background:**

*Pseudomonas aeruginosa* airway infections are a major cause of morbidity and mortality in patients with cystic fibrosis (CF). Azithromycin improves the related clinical outcomes, but its mechanisms of action remain poorly understood. We tested the hypothesis that azithromycin downregulates *P. aeruginosa*-induced pro-inflammatory responses by modifying release of bacterial proteins.

**Methods:**

We monitored inflammatory markers in lungs of CF mutant mice and their littermate controls in response to conditioned media (CM) collected from the reference *P. aeruginosa* PAO1 strain cultured in the presence or in the absence of azithromycin. A mass spectrometry-based proteomic approach was applied to examine whether the macrolide elicits a differential release of bacterial proteins.

**Results:**

CM collected from azithromycin-untreated PAO1 cultures induced powerful pro-inflammatory neutrophil-dominated responses. Azithromycin attenuated the responses, mainly of macrophage chemoattractant protein-1, tumor necrosis factor-α, and interferon-γ, in CF but not in wild-type mice. Proteomic analysis showed that azithromycin upregulated an array of bacterial proteins including those associated with regulation of immune functions and with repair and resolution of inflammatory responses like the chaperone DnaK and the *S*-adenosylmethionine synthase, while it downregulated the extracellular heme acquisition protein HasA and the catalytic enzyme lysylendopeptidase.

**Conclusion:**

Supernatants collected from cultures of the bacterial strain PAO1 represent a novel experimental model to trigger *in vivo* lung inflammatory responses that should be closer to those obtained with live bacteria, but without bacterial infection. Combined with a bactericidal effect, complex regulation of bacterial innate immune and metabolic factors released in the cultured medium by the action of the macrolide can contribute to its anti-inflammatory effects.

## Introduction

Recurrent endobronchial infections by the opportunistic Gram-negative bacterium *Pseudomonas aeruginosa* are associated with high morbidity and mortality in cystic fibrosis (CF). The virulence of *P. aeruginosa* stems from multiple factors such as its ability to release bacterial toxins, to develop antibiotic resistance, to form biofilm, to employ a range of cell-to-cell communication signals through quorum-sensing systems, and to acquire a mucoid phenotype; all these attributes render the microbe resistant to the innate and acquired immunologic defenses of the host (1, 2). Colonization of CF lungs with *P. aeruginosa* increases rates of lung function decline, worsens the prognosis of the disease, and is a significant predictor of mortality (3, 4). Active treatment of lung disease is a cornerstone of CF management. This may include anti-inflammatory therapy approaches in combination with antibiotic therapies (5–7) to circumvent the unbalanced exaggerated pro-inflammatory and decreased anti-inflammatory reactions. These have been considered in CF either as intrinsic or as a response to present or recent infection (8–11). Treatment with azithromycin, a macrolide antibiotic structurally modified from erythromycin, has been reported to attenuate symptoms in CF patients, resulting in significant clinical improvement in lung function with reduction in pulmonary exacerbations and fewer courses of antibiotic use (12–16). Regulation of bacterial virulence factors (17) and anti-inflammatory effects (18) of the macrolide have been postulated, but its underlying mechanisms of action are still under debate.

We hypothesized that azithromycin modulates *P. aeruginosa*-induced lung inflammation in CF by modifying release of bacterial products. After validating the model of conditioned media (CM) collected from the reference *P. aeruginosa* PAO1 strain to induce lung inflammation, we examined the influence of pre-treating bacterial cultures with azithromycin on lung pro-inflammatory markers in CF mice homozygous for the F508del mutation (F508del-CF) and in wild-type mice. CMs collected from PAO1 were analyzed by a mass spectrometry (MS)-based proteomic approach to characterize the differentially released bacterial proteins and to identify possible targets of the macrolide.

## Materials and Methods

### Animal Model

Female CF mice homozygous for the F508del-CFTR mutation built in the 129/FVB outbred background (Cftr^tm1Eur^) (19), and their normal homozygous wild-type littermates were studied. Mouse age ranged from 10 to 16 weeks and their weights between 20 and 30 g. Animals were housed following European recommendations (20) and regulations (CEE no. 86/609). The experimental protocol was approved by the local Ethics Committee for animal research (2013/UCL/MD/012). To prevent intestinal obstruction in CF mice, Movicol (55.24 g/L) was administered in demineralized acidified drinking water. The genotype of each animal was checked at 21 days of age, as described previously (18).

### Collection of Bacterial CM

The reference (ATCC 15692) *P. aeruginosa* PAO1 was chosen as it represents the most commonly used strain in research on this ubiquitous opportunistic Gram-negative microorganism. Collection of bacterial CM was obtained, as described previously (21). Briefly, the strain was seeded onto Difco™ Tryptic Soy Agar (TSA; Becton Dickinson and Co., Le Point de Claix, France) plates and allowed to grow overnight at 37°C. After seeding onto modified Vogel–Bonner medium (MVBM), cultures were incubated overnight under continuous agitation. The next day, *P. aeruginosa* bacteria were diluted in MVBM to reach 10^−8^ CFU/mL (optical density, OD of 0.1 at 600 nm) and incubated at 37°C for 16 h in the absence or in the presence of 8 mg/L azithromycin. This concentration is in the subminimum inhibitory range for *P. aeruginosa* and consistent with the concentration found in lung of patients treated with multiple azithromycin doses of 500 and 1000 mg daily (22). *P. aeruginosa* cultures, normalized to 0.2 OD at 600 nm, were collected by centrifugation (7000 × *g*, 30 min, 4°C) followed by filtration (0.22 μm filter) to remove any remaining bacteria. CMs were concentrated 17-fold using Amicon^®^ Ultra-15 30K NMWL centrifugal filter devices (Millipore Corporation, Bedford, MA, USA) precoated with 10 mg/mL bovine serum albumin (BSA, Sigma Chemical, St. Louis, MO, USA). CM samples were ultra-centrifuged (70,000 × *g*, 1 h, 4°C), filtered on gel (PD-10 Desalting columns; GE Healthcare Bio-Sciences AB, Uppsala, Sweden), and finally filtered on 0.22 μm filter and stored at −80°C.

### Induction of Lung Inflammation by Exposure to LPS or CM

Weight-matched F508del-CF and normal homozygous wild-type mice were anesthetized with an intraperitoneal (i.p.) injection of a mixture of 100 mg/kg ketamine (Parke-Davis, Ann Arbor, MI, USA) and 15 mg/kg xylazine (Bayer, Leverkusen, Germany). As a reference model of induction of acute lung inflammation, a standard dose of LPS (Sigma Chemical, St Louis, MO, USA; 50 μg/25 g body weight in 50 μL saline) was instilled into the trachea through the mouth, using a laryngoscope and a fine pipette tip. To track, at a single-time point after LPS exposure, both the early cytokine responses and the late cell infiltrate responses, a validated combined protocol (23) using two consecutive doses (50 and 100 μg/25 g body weight in 50 μL saline) with a 21-h interval between doses was applied, with sampling taking place 3 h after the last dose. In control experiments, sterile saline was used as solution for endotracheal instillation.

A volume of 50 μL CM collected from PAO1 was instilled into the mouse trachea. In order to select the optimal doses of CM, serial dilutions were tested at selected time points after endotracheal instillation. As for the LPS combined protocol, two CM doses were applied at an interval of 21 h with sampling 3 h after the last instillation.

### Bronchoalveolar Lavage

At selected time points after the last endotracheal instillation of NaCl, LPS, or CM, the mice were killed by i.p. injection of 20 mg sodium pentobarbital (Abbott, Chicago, IL, USA). Bronchoalveolar lavage (BAL) was then performed by cannulating the trachea and lavaging with 1.5 mL sterile saline, as described previously (18). The BAL fluid was centrifuged (250 × *g*, 10 min, 4°C) and the supernatant was aliquoted and stored at −80°C for further biochemical measurements unless otherwise stated. Cell pellets were resuspended in saline to count total cells in fresh preparations using a Burker cell. Cells were spread on microscope slides by cytospin centrifugation (Cytospin 3, Shandon, Pittsburgh, PA, USA). Differential cell counts (at least 200) were performed after fixation with methanol and staining using the Diff Quick method (Dade, Brussels, Belgium).

### Biochemical Analyses

The activity of the cytoplasmic enzyme lactate dehydrogenase (LDH) in fresh BAL fluid was evaluated spectrophotometrically based on the catalytic oxidation of l-lactate to pyruvate with concomitant reduction of nicotinamide adenine dinucleotide (NAD). The enzyme activity was measured by monitoring NADH formation at 340 nm. Linearity of the test ranges from 20 to 3800 U/L.

Total protein content in fresh BAL fluid was analyzed by a dye-binding colorimetric assay using an automated pyrogallol red-molybdate-protein complex method (SYNCHRON LX^®^, Beckman Coulter, Brea, CA, USA). Color change (from red to blue under test conditions when complexed with proteins) was measured as absorbance change at 600 nm. The limit of detection was from 0.06 to 3.00 g/L.

The master cytokines [tumor necrosis factor (TNF)-α, interleukin (IL)-1β, and IL-6]; the chemokines [keratinocyte chemoattractant (KC), macrophage chemoattractant protein (MCP)-1, and mouse macrophage inflammatory protein (MIP)-2]; IL-17 and the Th-1-related cytokine, interferon (IFN)-γ, were simultaneously monitored in BAL fluid following the manufacturers’ protocol using a Multiplex assay (Merck Millipore, Darmstadt, Germany). The lower limit of detection for all cyto/chemokines was 3.2 pg/mL. In experiments conducted to validate the CM protocol, the two master cytokines TNF-α (BD Pharmigen; San Diego, CA, USA) and IL-1β (R&D Systems; Minneapolis, MN, USA) were monitored in BAL fluid using a standard sandwich enzyme-linked immunosorbent assay (ELISA). Their lower limit of detection was 15.6 pg/mL.

All biochemical analyses were performed in duplicates.

### Quantification of LPS

The amounts of LPS in samples used for endotracheal instillation were measured using a chromogenic signal generated in the presence of the endotoxin by a limulus amebocyte lysate (LAL) Pierce chromogenic quantitation kit assay (ThermoFisher Scientific, Rockford, IL, USA). The reaction was measured on a microplate absorbance reader at 405 nm following the manufacturer’s protocol. A standard curve was created using LPS from *P aeruginosa* (1 mg/mL in NaCl). The microplate was incubated at 37°C for 10 min, and the chromogenic substrate solution was then added. The reaction was stopped by adding 25% acetic acid within 6 min when a yellow color appeared. Values are expressed as endotoxin units (EU) with 1 EU/mL corresponding to 1 ng endotoxin/mL of solution. The limit of detection is 0.5 EU/mL. Quantification of LPS was performed in triplicates.

### Proteomic Analysis

#### Tryptic Digestion

Equal amounts of non-diluted CM (1 mL), collected from PAO1 cultures in the presence or in the absence of azithromycin, were concentrated to 100 μL. The total protein content was quantified by SPN™-Protein Assay (G-Biosciences, St Louis, MO, USA). The 50 μg protein per sample was treated with RapiGest™ SF at 0.2% (w/v) (Waters Corporation, Milford, MA, USA). After incubation at 100°C for 5 min, samples were cooled to room temperature and digested with trypsin (Sequencing Grade Modified Trypsin, Promega, Madison, WI, USA) at an enzyme/substrate ratio of about 1:50 (w/w). Samples were then incubated at 37°C overnight and another aliquot of trypsin [1:100 (w/w)] was added to samples further incubated at 37°C for 4 h. The enzymatic reaction was stopped by acidification with 0.5% trifluoroacetic acid (Sigma-Aldrich) followed by incubation at 37°C for 45 min and centrifugation at 13,000 × *g* for 10 min. Samples were desalted by PepClean C-18 spin columns (Pierce Biothecnology Inc., Rockford, IL, USA), concentrated in a SpeedVac (Savant Instruments Farmingdale, NY, USA) at 60°C, and finally resuspended in 0.1% formic acid (Sigma-Aldrich Inc., St. Louis, MO, USA).

#### Mass Spectrometry-Based Proteomic Analysis

Trypsin-digested samples (*n* = 3 replicates per condition) were analyzed by multidimensional protein identification technology (MudPIT) based on two dimensional micro-liquid chromatography coupled with tandem MS. Briefly, 3 μL of the digested peptide mixture were loaded by means of an autosampler (Suveyor AS Thermo) onto a strong cation exchange column (PolySULFOETHYL A™ Capillary, 0.3 mm i.d. × 100 mm, and 5 μm, PolyLC Inc., Columbia, MD, USA) and then eluted using eight steps of increasing ammonium chloride concentrations (0, 20, 40, 80, 120, 200, 400, and 700 mM). Eluted peptides, obtained by each salt step, were first captured in turn onto two peptide traps (Zorbax 300 SB C-18, 5 μm, and 0.3 mm i.d. × 5 mm, Agilent technologies, Santa Clara, CA, USA) mounted on a 10-port valve, for concentration and desalting, and subsequently loaded on a reversed phase C-18 column (BioBasic-18, 0.180 mm i.d. × 100 mm, and 5 μm, Thermo Electron Corporation, Bellofonte, PA, USA) for separation with an acetonitrile gradient. The flow rate on C-18 column was 1 μL/min. Peptides eluted from the C-18 column were directly analyzed with a linear ion trap LTQ mass spectrometer (Thermo Fisher) equipped with a nanospray ion source. The spray capillary voltage was set at 1.5 kV, while the ion transfer capillary temperature was held at 185°C. Full mass spectra were acquired in positive mode and over a 400–2000 *m*/*z* range, followed by five MS/MS events sequentially generated in a data-dependent manner on the five most-intense ions selected from the full MS spectrum, using dynamic exclusion for MS/MS analysis (collision energy 35%).

#### Proteomics Data Processing

The experimental MS/MS spectra produced by MudPIT were matched against the *in silico* tryptic peptide sequences of the *P. aeruginosa* protein database (5679 protein sequences) retrieved from UNIPROT database^1^ on May 2015. Data processing was performed by Discoverer 2.0 software, based on SEQUEST HT algorithm (24). Peptide and protein assignment was made according to specific guidelines (25). The following criteria were used for peptide identification: parent and fragment mass tolerance of 1 and 0.6 Da, respectively, and missed cleavage sites per peptide of 2. Matches between spectra were only retained if they had a minimum Xcorr of 2.0 for +1, 2.5 for +2, and 3.5 for +3 charge state, respectively, protein rank was fixed to 1, while peptide confidence was set to “*high*.” In addition, the false discovery rate (FDR) was set to ≤3%. Based on a direct correlation between the spectral count (SpC) and the relative protein abundance, identified proteins were semi-quantitatively compared by label-free approaches (26). Specifically, protein lists were processed by means of linear discriminant analysis (LDA) (27) using a common covariance matrix for all groups and the Mahalanobis distance (28) from each point to each group’s multivariate mean. To select proteins discriminating the analyzed *P. aeruginosa* conditions, we considered those with smallest *p*-value (≤0.05), while *F* ratio resulted ≥9. The average SpC (aSpC) values of proteins selected by LDA were pairwise compared by Dave and DCI indices of MAProMa. In between-group comparisons, each protein resulted with a Dave value >|0.3|. Finally, proteins selected by LDA, Dave, and DCI indices were evaluated by unsupervised learning methods, such as hierarchical clustering (HC) (29), using in-house R-scripts, based on XlsReadWrite, clue, and clValid library^2^; *Ward*’s method and *Euclidean* distance metric were applied.

### Statistics

Statistical analysis was performed using JMP12 software (SAS Institute, Cary, NC, USA). Data are expressed as mean ± SD, where *n* refers to the number of animals per group. Between-group comparisons were evaluated assuming normal distributions by ANOVA with *post hoc* comparisons made by using Student’s *t*-test or Tukey–Kramer honestly significant difference test for two or more than two *x*-levels, respectively. Null hypothesis was rejected at *p* < 0.05.

## Results

Endotracheal instillation of CM collected from *P. aeruginosa* PAO1 strain was well tolerated by wild-type and F508del-CF mice, and no mortality was observed. As NaCl instillation does not influence any monitored inflammatory marker (18, 23), values obtained under saline-treated conditions in wild-type or in F508del-CF mice were considered as control reference values for the corresponding genotype group.

### CM from *Pseudomonas* Induces Strong Pro-inflammatory Responses

To validate the CM collected from PAO1 cultures as a model to study *in vivo* inflammatory responses, a first series of experiments was conducted to select its optimal dilution either at 3 h after mouse instillation, to evaluate early cytokine release responses, or at 24 h after mouse instillation, to evaluate late cellular infiltrate responses (18). We then measured release of the representative TNF-α master cytokine and cell counts, 3 and 24 h, respectively, after instillation of CM diluted (0, 3, 10, and 30 times) with saline. As a control, the effects were compared with those analyzed at the same time point after a single standard dose of LPS known to induce powerful neutrophil-dominated pro-inflammatory responses (18, 23).

**Figure 1:**
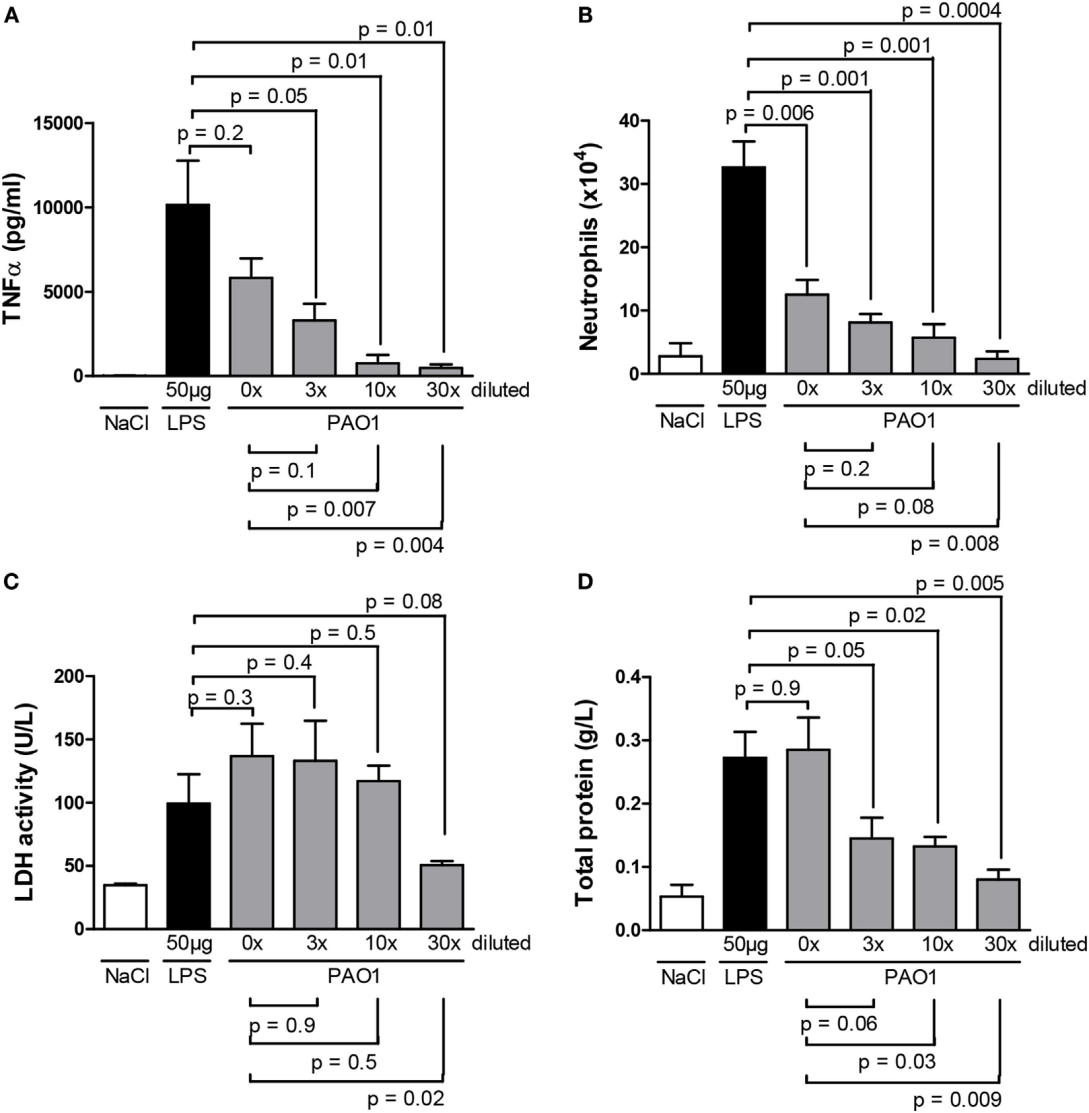
**Master cytokine [(A) TNF-α], neutrophil infiltrate (B), markers of cytotoxicity [(C) lactate dehydrogenase, (D) total protein] in the bronchoalveolar lavage of wild-type mice 3 h (A) or 24 h (B–D) after a single endotracheal instillation with serial dilutions (0, 3, 10, and 30 times) of CM collected from PAO1 cultures, in comparison with a standard LPS dose (50 μg/mouse) at the same time point or with saline**. Data illustrate one of three representative independent experiments. Values are means ± SD for four animals per group. *p*-Values denote levels of significance of comparisons between PAO1 dilutions and LPS (supra) or non-diluted PAO1 samples (infra).

As illustrated in Figure 1A, no significant difference was observed on TNF-α obtained from BAL samples of mice treated with non-diluted PAO1 or with LPS; a dilution-dependent effect was observed from a dilution factor of 3 onward. These findings indicate that the non-diluted CM from PAO1 is as powerful as LPS to trigger pro-inflammatory responses. However, the cellular component of the inflammatory response, characterized by a dominant neutrophil infiltrate, was significantly less in non-diluted PAO1 samples than in LPS samples (Figure 1B), suggesting a possible cytotoxic effect of CM. Compared to the non-diluted CM sample, a minor non-significant reduction of neutrophil count was observed with the dilution factor 3, while the cell count was reduced to about 50% or even to basal levels with higher dilution factors.

To better characterize the possible cytotoxic effect of CM, we monitored, in BAL fluid obtained 24 h after a single dose of serial CM dilutions, LDH activity as a marker of cytotoxicity, and total protein content as a maker of vascular permeability. LDH activity (Figure 1C) and total protein content (Figure 1D) were reduced with higher CM dilutions, suggesting a dilution-dependent cytotoxic effect of CM. However, as at the 3× dilution, near maximal cell counts were reached (Figure 1B) with an apparent reduction of cytotoxicity (Figure 1D), the 3× CM dilution was selected for the 24-h time point in further experiments.

**Figure 2:**
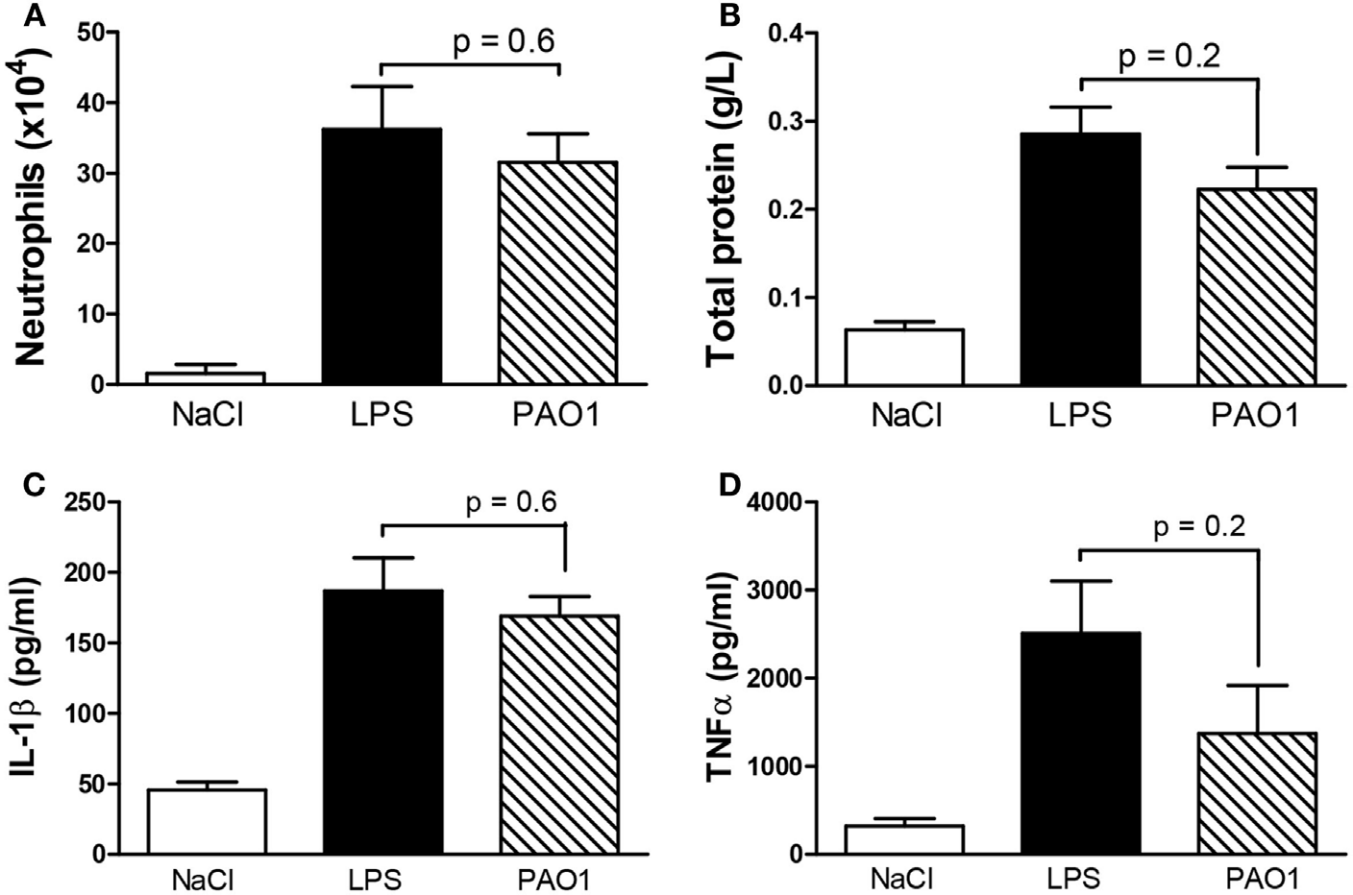
**Master cytokines [(A) TNF-α; (B) IL-1β], neutrophil infiltrate (C), and total protein content as a marker of cytotoxicity (D) in the bronchoalveolar lavage of wild-type mice after endotracheal instillation with the combined two-dose protocol of CM collected from PAO1 cultures, in comparison with the combined two-dose LPS protocol or saline**. Combined CM protocol consisted in a first dose diluted three times followed by a second non-diluted dose. Combined LPS protocol consisted in a first dose, 50 μg and a second dose, 100 μg. Interval between doses (50 μL) in both protocols was 21 h and sampling 3 h after the second dose. Data illustrate one of three representative independent experiments. Values are means ± SD for four animals per group, except for neutrophil count and total protein in the NaCl group (*n* = 3). *p*-Values denote levels of significance of comparisons between PAO1 and LPS.

To allow reducing the number of mice tested and tracking both early cytokine release responses and late cellular infiltrate responses in the same samples, a combined CM protocol was designed in which the second non-diluted CM solution was applied 3 h before sampling. In further experiments, the combined CM protocol consisted in the first dose diluted three times (shown to reduce possible cytotoxicity effects), followed by the second non-diluted dose (shown to induce maximal cytokine release responses). To validate the CM combined protocol, we compared values of master cytokines (IL-1β and TNF-α), cell counts, and markers of cytotoxicity in BAL samples obtained using either the CM combined protocol or the validated LPS combined protocol. The latter consisted in two subsequent LPS doses (50 and 100 μg) applied at a 21-h interval with sampling 3 h after the latter and larger dose (23). We confirmed here the efficacy of the LPS combined protocol to induce both early cytokine and late cellular responses (Figures 2A–C). We showed that the CM combined protocol was as effective as the LPS combined protocol to induce pro-inflammatory responses without significant differences in cytotoxicity. Indeed, similar values were obtained for total protein contents in BAL fluid (Figure 2D) and LDH activity (data not shown) in the two protocols. Altogether, these data indicate that, as the LPS model, CM has a strong neutrophil-chemoattractant effect. Thus, the combined CM protocol is a valuable model to study both early (cytokine release) and late (cellular infiltrate) components of lung pro-inflammatory responses without apparent cytotoxic effects.

### Pro-inflammatory Effects of CM Are Not Mainly Related to LPS

**Figure 3:**
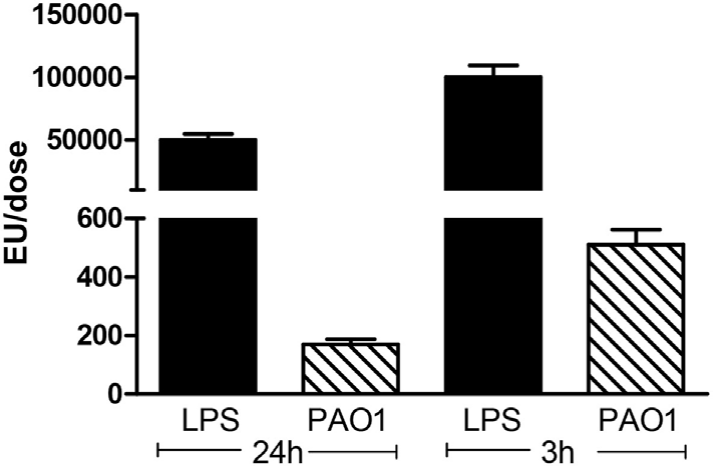
**Quantification of LPS in CM obtained from PAO1 cultures, in comparison with LPS solutions**. CM samples applied 24 or 3 h before sampling were diluted three times or not diluted. LPS dose applied 24 or 3 h before sampling was 50 or 100 μg. Data illustrate one of three representative independent experiments performed in triplicate. Values are means ± SD for three samples per group.

To analyze whether the pro-inflammatory effects observed with CM collected from PAO1 were dependent on LPS released into the culture medium, the endotoxin was quantified in CM and in LPS solutions used for endotracheal instillation. Compared to LPS, the CM doses applied 24 and 3 h before sampling were about 300 times and 200 times lower (Figure 3). These findings indicate that the strong pro-inflammatory effects of CM from PAO1 are not mainly related to LPS but rather due to non-LPS-related bacterial products released in the supernatant of cultures.

### Pretreatment of Bacterial Cultures with Azithromycin Reduces Lung Pro-inflammatory Responses in CF Mice

To test the influence of azithromycin on the magnitude of lung inflammatory responses induced by CM collected from PAO1 cultures treated or not treated with the macrolide, F508del-CF, and wild-type mice were instilled using the combined CM protocol.

**Figure 4:**
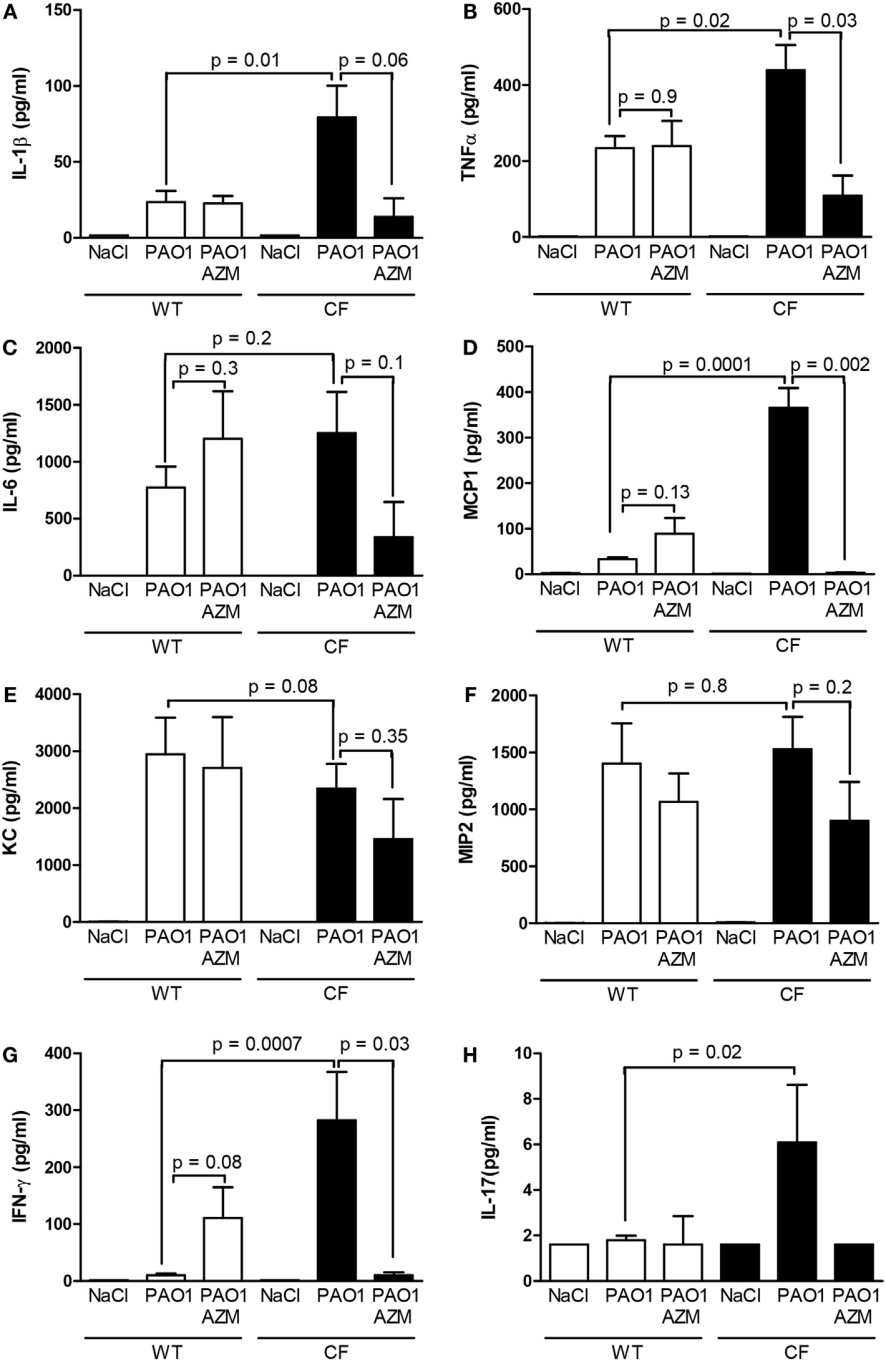
**Cytokines (A–H) in the bronchoalveolar lavage of wild-type and CF mice after endotracheal instillation with the combined two-dose protocol of CM collected from the PAO1 strain cultured in the absence (PAO1) or in the presence of azithromycin (PAO1 + AZM), in comparison with saline**. Data illustrate one of three representative independent experiments. Values are means ± SD for seven animals in the wild-type group and three animals in the CF group. *p*-Values are indicated for comparisons between responses according to the genotype and to the presence of the macrolide.

Under control NaCl conditions, no significant differences were found in the lung inflammatory phenotype between F508del-CF and wild-type mice (Figures 4A–H). The mean (±SD) total cell count in BAL was 2.3 (±0.3) × 10^4^ in the wild-type group and 2.4 (±0.6) × 10^4^ in the CF group. No genotype-associated differences were noted in the proportions of cell populations, i.e., neutrophils and macrophages (data not shown), in the expression of master cytokines (IL-1β and TNF-α IL-6), chemokines (MIP-2, KC, and MCP-1), IL-17 and the Th1-related cytokine, and IFN-γ, in BAL (Figures 4A–H).

After exposure to CM, the release of almost all cyto/chemokines (except KC, MIP-2, and IL-6) was increased in F508del-CF compared to wild-type mice (Figures 4A–H). Interestingly, CM-induced IL-17 release was only triggered in the CF group and by CM PAO1 without azithromycin (Figure 4H). The observation is in line with clinical findings, suggesting that T helper 17 pathway may contribute to the CF lung pathology (30). In wild-type mice, no significant effect of azithromycin was detected on any of the cyto/chemokines analyzed. In CF mice, a significant effect of the macrolide was observed in all cyto/chemokines that were overexpressed in response to the CM exposure, in particular, MCP1, TNF-α, and IFN-γ. These data indicate that treating bacterial cultures with azithromycin attenuates the ability of the culture media to induce lung inflammatory responses. The attenuating effect of azithromycin on the inflammatory responses to CM exposure seems to be genotype-related: CF mice are more recipient than wild-type mice. We could postulate that the macrolide exerts its action in CF lung disease, at least partly, by inhibiting the NFκB pathway and/or the Th17 pathway.

### Pretreatment of Bacterial Cultures with Azithromycin Promotes Release of Bacterial Proteins Possibly Reducing Lung Pro-inflammatory Responses in CF Mice

**Figure 5:**
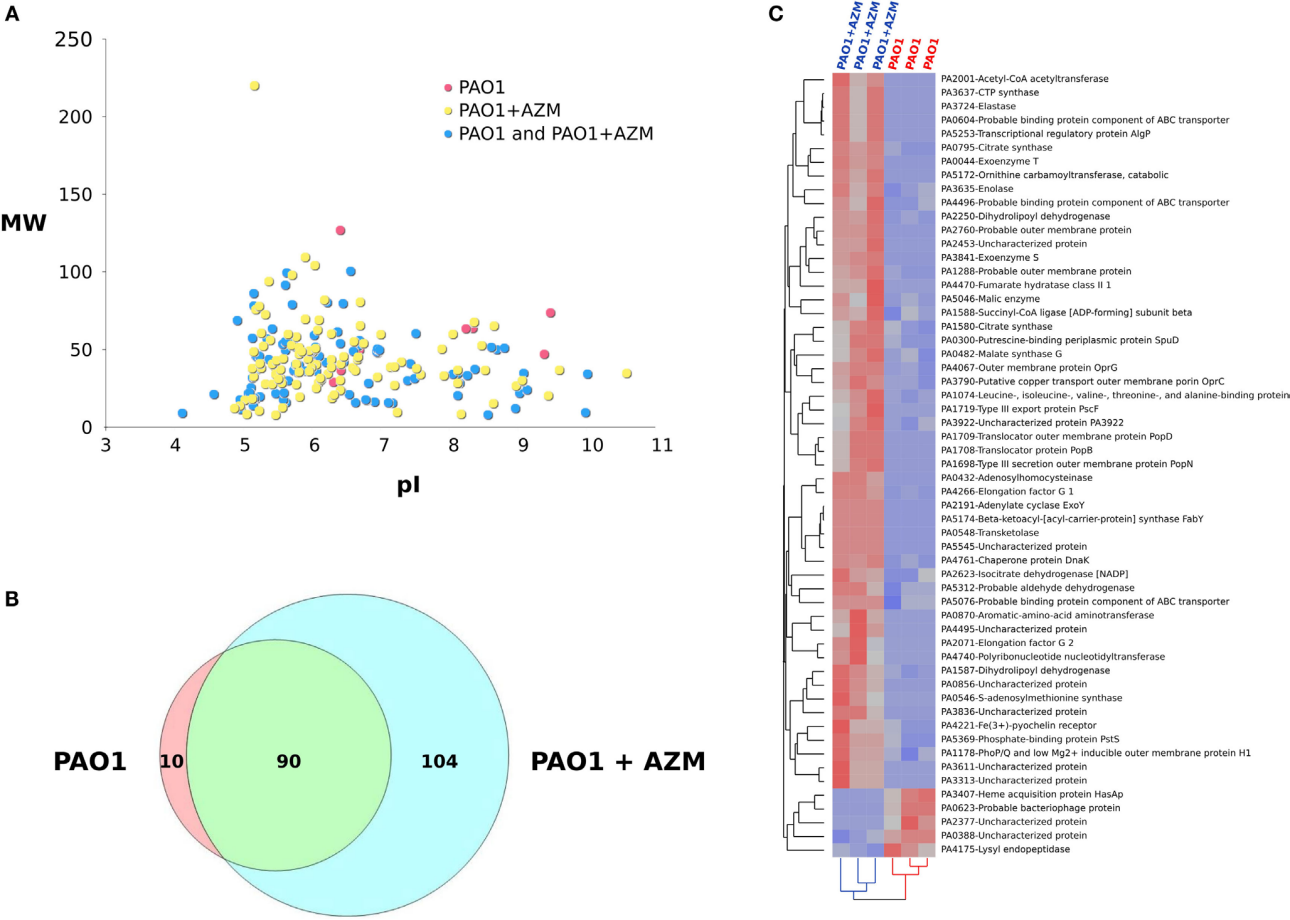
**Influence of azithromycin on proteins released by *Pseudomonas aeruginosa* when cultured in the absence (PAO1) or in the presence of the macrolide (PAO1 + AZM)**. **(A)** Virtual 2D map (molecular weight, MW vs. isoelectric point, pI) of protein identified by means of MudPIT approach by analyzing CM from PAO1 (*n* = 3) and PAO1 + AZM (*n* = 3); red spots: proteins specifically identified in the CM of PAO1, yellow spots: proteins specifically identified in the CM of PAO1 + AZM, and blue spots: proteins identified in the CM of both PAO1 and PAO1 + AZM. **(B)** Venn diagram of proteins identified in the CM from PAO1 and PAO1 + AZM. **(C)** Hierarchical clustering of the CMs derived from PAO1 and PAO1 + AZM; heat map shows the spectral count of the proteins found differentially expressed when comparing PAO1 and PAO1 + AZM.

CM collected from PAO1 cultured in the presence or in the absence of azithromycin were next analyzed by means of MudPIT approach to identify candidate bacterial products as possible targets of the macrolide effect. Globally, following six MudPIT runs, 204 proteins were identified (Figure 5A, Data sheet S1 in Supplementary Material) and about 75% resulted with a total SpC > 2 (Table S1 in Supplementary Material). Of note, proteins released by CM cultured in the presence of azithromycin were twice as numerous as those released by the non-treated CM (Figure 5B). In this scenario, the semi-quantitative comparison between proteins released by *P. aeruginosa* in the presence or in the absence of the macrolide allowed identification of 57 differentially expressed proteins the expression of which changed in a reproducible way in all samples analyzed (Figure 5C). In particular, five proteins (positive Dave value) were downregulated in the CM collected from azithromycin-treated cultures, while 52 proteins were upregulated (negative Dave value) in CM from azithromycin-treated cultures (Table S2 in Supplementary Material). Most of the differentially expressed proteins were involved in transport and metabolic processes.

Among proteins possibly attenuating virulence factors of *P. aeruginosa* pathogenesis, we found that treatment with azithromycin downregulated the catalytic enzyme lysyl endopeptidase (prpL-PA4175) and the extracellular heme acquisition protein HasAp (hasAp-PA3704), an iron-regulated extracellular protein that captures free or hemoglobin-bound heme. The treatment upregulated *S*-adenosylmethionine synthase (metK-PA0546), involved in generation of the nucleoside 5′-methylthioadenosine (MTA) that has multiple anti-inflammatory properties (31, 32), and the chaperone protein DnaK (dnaK-PA4761), recognized as a modulator of macrophage phenotype able to drive polarization of macrophages to a M2 profile (33) involved in repair and resolution of inflammatory responses (34).

Many other proteins were found to be upregulated under the influence of azithromycin treatment. Some are cytotoxic exoproducts such as exoenzyme S (exoS-PA3841), exoenzyme T (exoT-PA0044), transporters, or metabolic (co)factors such as adenylate cyclase (ExoY-PA2191), elastase (lasB-PA3724), Fe(3^+^)-pyochelin receptor (fptA-PA4221), and transcriptional regulatory protein AlgP (algP-PA5253). In addition, dihydrolipoyl dehydrogenases (lpdG-PA1587 and lpdV-PA2250), protecting the bacterial pathogen from the action of the terminal complement pathway, were found upregulated in the presence of the macrolide. Altogether, these findings show that pretreatment of *Pseudomonas* cultures with azithromycin promotes a differential release in the culture medium of a combination of bacterial proteins possibly reducing lung pro-inflammatory responses in CF mice.

### Azithromycin Has a Bactericidal Effect on the PAO1 Strain of *Pseudomonas* in Culture

**Figure 6:**
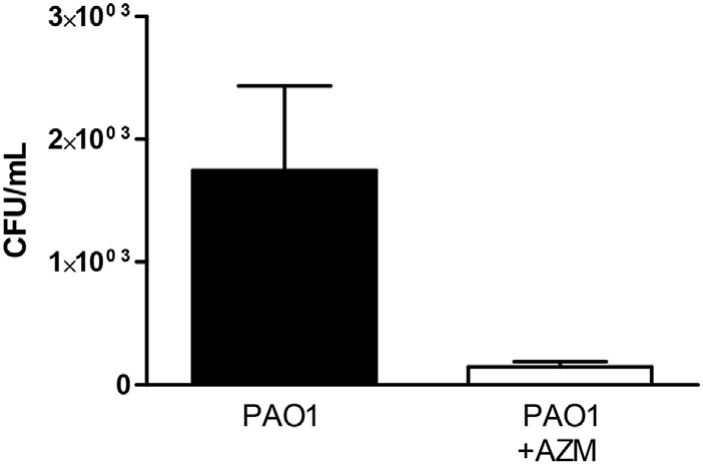
**Counts of colony-forming units (CFU) in conditioned medium obtained from PAO1 cultures grown in the presence or in the absence of azithromycin (AZM)**. Data are expressed as mean ± SD for six independent experiments performed in duplicate (**p < 0.01).

To get deeper insight into the mechanism of the reduction by azithromycin of the pro-inflammatory effect of *Pseudomonas*, we evaluated the effect of the macrolide on the number of colony-forming units (CFU) in the culture medium. We observed (Figure 6) that azithromycin reduced counts of CFU by over 60 times, indicating a bactericidal effect on the PAO1 strain.

## Discussion

The present work was designed to test the hypothesis that azithromycin modulates lung inflammation by modifying the set of bacterial proteins released in the cultured medium. The growing interest in macrolide antibiotics as beneficial agents in CF followed the success of long-term erythromycin in the treatment of diffuse pan-bronchiolitis, a condition that exhibits striking similarities to CF (35, 36). Azithromycin, a macrolide structurally modified from erythromycin, is an “unconventional” antibacterial drug for which a growing body of evidence brought about by clinical experience (37) and clinical trials (12–16) has demonstrated beneficial in CF. Macrolide antibiotics inhibit bacterial growth by binding to the 23S rRNA in the 50S subunit of the bacterial ribosome, preventing the transfer of tRNA from the A to the P site of the ribosome (38, 39). How this mechanism translates into clinical efficacy is still a matter of debate. Numerous studies have addressed the issue focusing on both sides of the host/pathogen equation. Interestingly, it has been reported that azithromycin attenuates pro-inflammatory responses (18, 23) and promotes macrophage phagocytic activity properties (40) in the treatment of infections involving azithromycin-resistant pathogens, such as the opportunistic *P. aeruginosa* (41). This suggests that anti-inflammatory and anti-virulence properties of azithromycin contribute to its beneficial effects. As this is a central issue in the management of CF, better understanding of its mechanism of action is highly relevant.

We showed here that CM collected from the reference bacterial strain PAO1 represents a novel experimental model to study *in vivo* sterile inflammatory responses. CM from *Pseudomonas* cultures was shown to be as powerful a neutrophil-dominated pro-inflammatory model as LPS. Beyond its much lower LPS content, the large set of released bacterial proteins, demonstrated by proteomic analyses, has the additional advantage of triggering reactions that should be closer to those obtained with microbial models using live bacteria, but without bacterial infection. This is a major concern for managers of animal facilities housing immunodeprived or CF mouse colonies. Moreover, exposure to CM from PAO1 allows investigating inflammatory responses independently of clearance of bacteria, defective in CF. Indeed, it has been proposed that CFTR is a cellular receptor for binding, endocyting, and clearing *P. aeruginosa* from the normal lung (42–44). Even though it also allows studying responses that are independent of interactions of bacteria with epithelial cells, a drawback of the LPS model is that non-mucoid to mucoid conversion of *Pseudomonas*, a poor prognosticator in CF, is associated with a loss of high molecular weight LPS species (45).

We confirmed here that the CF mouse model homozygous for the most common F508del-CFTR mutation displays exaggerated inflammatory responses (18, 23). We showed that responses to azithromycin seemed to be dependent on the CF genotype. Indeed, our data showed that in CF mutants, pretreatment of *Pseudomonas* cultures with azithromycin reduced the release of all cyto/chemokines that overresponded to exposure to CM.

The data we present here indicate that azithromycin releases a differential set of extracellular and intracellular bacterial proteins which may trigger anti-inflammatory reactions in the host. Using high-throughput screening procedures, the bacterial products were identified as taking part in a myriad of processes, such as regulation of immune responses, membrane transporter mechanisms, or as (co)factors, in metabolic processes. At least some of these products may play critical roles in attenuating the virulence of the bacteria. Among the few downregulated proteins identified in CM collected from bacterial cultures treated with azithromycin, lysylendopeptidase, and heme acquisition protein, HasAp can be highlighted as targets of the anti-virulent effect of the macrolide. Lysylendopeptidase is a catalytic enzyme that specifically cleaves peptide bonds at the carboxyl side of lysine residues (46). It can be assumed that its downregulation exerts protective, anti-proteolytic effects on host tissues. Heme acquisition protein HasAp is a bacterial iron-regulated extracellular heme-binding protein that is required for *P. aeruginosa* utilization of hemoglobin iron (47). It is known that the ability of *P. aeruginosa* to cause chronic infections in patients with CF also depends on iron-scavenging systems (48). Downregulation of heme acquisition protein HasAp is expected to negatively impact on bacterial virulence by reducing availability of hemoglobin to the bacterium at the local host microenvironment level. Upregulation of DnaK and the *S*-adenosylmethionine synthase might also negatively impact on the virulence of the bacterium. The former is the major bacterial counterpart of the heat shock protein 70, found to act as an important immunomodulator of monocytes, dendritic cells (49–51), and macrophage responses. It has been recognized as being capable of polarizing macrophages to the M2 phenotype by inducing higher arginase I activity, IL-10 production, and FIZZ1 and Ym1 expression (33). The latter generates *S*-adenosylmethionine which is converted into 5′-methylthioadenosine (MTA) (52), which has anti-inflammatory properties (33). Based on the immunomodulating effect of DnaK, by driving M2 macrophage polarization, and on the anti-inflammatory effect of MTA on multiple cytokines, it can be assumed that treatment of *Pseudomonas* cultures with azithromycin induces a tolerogenic phenotype linked to the *in vivo* anti-inflammatory effects we observed. The increased release of MTA, with anti-inflammatory properties, can be beneficial to the host responses.

It is more challenging to integrate data regarding expression of exoenzymes S and T and of other proteins, such as PopD and PopN, comprising the type III secretion system which is a key virulence strategy found in Gram-negative bacteria. They were found to be upregulated in azithromycin-treated bacterial cultures. In pathogenic bacteria, effector proteins composing the type III secretion system are secreted directly into the cytosol of host cells as a strategy to facilitate bacterial pathogenesis (53). ExoS and ExoT are bifunctional toxins with GTPase-activating protein and ADP-ribosyltransferase activities (54). PopD and PopN are hydrophobic proteins required for bacterial translocation of Exo S and T (55). Studies have shown that the type III secretion system specifically interferes with host cell signal transduction as well as other cellular processes to promote mucosal barrier injury, deregulation of innate immune responses, and prevention of wound repair (56). In particular, ExoS appears to elicit a cytotoxic phenotype in cultured cells, while ExoT interferes with host cell phagocytic activity. Accordingly, intratracheal administration of ExoS resulted in neutrophil infiltration and in intense inflammatory responses correlating with pulmonary damage in animal models (57) and in patients with CF (58).

Interestingly, a study focusing on corneal infection with *P. aeruginosa* in mice revealed that the exoenzymes S and T are essential mediators promoting induction of neutrophil apoptosis *in vitro* and *in vivo* (59). This feature could contribute, at least partly, to the reduced pro-inflammatory responses we observed with the macrolide. It is noteworthy that the presence of exotoxins and associated translocon proteins do not necessarily lead to cell toxicity. As a matter of fact, a pro-myelocytic cell line (HL-60) and a pro-monocytic cell line (U937) were found to be resistant to toxin injections even in the presence of PopB/D associated with host cell plasma membranes (60). Examination of host cell components, such as cholesterol, actin cytoskeleton network, and signal transduction pathways, indicated that they can modulate the injection of exotoxins into eukaryotic cytoplasm. The authors (60) concluded that the efficacy of exotoxins delivered into host cells depends on regulation of translocation processes and suggested possible cross-talks between eukaryotic cell and the pathogen at the level of exotoxin translocation. Further studies are clearly required to identify the precise mechanisms governing this network of interactions.

Our data provide evidence of an inhibitory effect on bacterial growth has been documented, suggesting a bactericidal effect of the macrolide in the PAO1 strain. But in addition, we showed that the anti-inflammatory effect of azithromycin results from changes in a set of proteins released from different bacterial compartments. The underlying molecular basis of the latter seems to act *via* a complex regulation of the host microenvironment. Modulation of metabolic processes could include cell differentiation of monocytes/dendritic cells and macrophages to an anti-inflammatory (M2) phenotype that is believed to promote cell protection, regeneration, and plasticity. Additionally, downregulation of catalytic reactions and iron availability can impact on limiting virulence of *P. aeruginosa*. Targeting products released by the bacterium can help to identify strategies preventing lung damage in patients with CF. The recent development of more easily manageable murine models of lung inflammation (61) might facilitate the screening of anti-inflammatory compounds using experimental conditions that might better mimic the clinical setting (individual bacteria strains isolated form patients, for example).

## Conclusion

Supernatants collected from cultures of the reference bacterial strain PAO1 represent a novel experimental model to study *in vivo* sterile lung inflammatory responses in mice. Our combined two-dose protocol is well suited to track at the same time point the early (cytokine) and the late (cell infiltrate) components of the inflammatory responses. Pretreatment of *Pseudomonas* cultures with azithromycin attenuates the inflammatory overresponses in CF mice. By proteomic analyses, we showed that azithromycin releases a differential set of extracellular and intracellular bacterial proteins which may trigger anti-inflammatory reactions in the host. Our work also provides evidence of an inhibitory effect on bacterial growth, suggesting a bactericidal effect of the macrolide in the PAO1 strain.

## Author Contributions

Conceived and designed the experiments: GB, FH, NP, SN, BD, JH, PM, SM, DS, CS, PM, and TL. Performed experiments: NP, SM, and GB. Analyzed mouse data: NP, FH, and TL. Analyzed proteomic data: SM, DS, and CS. Wrote the paper: TL, NP, CS, and DS.

## Conflict of Interest Statement

The authors declare that the research was conducted in the absence of any commercial or financial relationships that could be construed as a potential conflict of interest.

## Footnotes

^1^www.uniprot.org/.

^2^http://cran.r-project.org.

## Supplementary Material

Table S1 **Proteins identified by analyzing supernatants of culture media collected from *Pseudomonas aeruginosa* cultured in the absence (PAO1; *n* = 3) or in the presence of pretreatment with azithromycin (PAO1 + AZM; *n* = 3)**. SpC, spectral count; aSPC, average spectral count; sSPC, spectral count sum; tot SpC, total spectral count.

Table S2 **Differentially expressed proteins by comparing the protein profiles of PAO1 and PAO1 + AZM CMs**. SpC, spectral count; aSpC, average spectral count; emPAI, exponentially modified protein abundance index; LDA, linear discriminant analysis, Dave, differential average; DCI, differential confidence index. Positive Dave value (red) indicate upregulation in PAO 1 CM, while negative Dave value (blue) indicate upregulation in PAO1 + AZM CM. For each protein, MW, pI, biological processes, cellular component, and molecular function are shown.

Data Sheet S1 **Peptides identified by analyzing supernatants of culture media (CM) collected from *Pseudomonas aeruginosa* cultured in the absence (PAO1) or in the presence of pretreatment with azithromycin (PAO1 + AZM)**. The file contains six data sheets corresponding to the MudPIT replicates analysis (rep 1–3) of CMs. For each analysis, Protein accession, annotated sequence, confidence, PSM ambiguity, protein groups, missed cleavages, charge, rank, *m*/*z* [Da], MH + [Da], DeltaM [ppm], Delta *m*/*z*, and Xcorr are shown.

## Abbreviations

CF, cystic fibrosis; CFTR, cystic fibrosis transmembrane conductance regulator; MudPIT, multidimensional protein identification technology.

## References

[1] Lyczak JB Cannon CL Pier GB. Lung infections associated with cystic fibrosis. Clin Microbiol Rev (2002) 15:194–222. 10.1128/CMR.15.2.194-222.2002 11932230 PMC118069

[2] Williams P. Quorum sensing, communication and cross-kingdom signalling in the bacterial world. Microbiology (2007) 153:3923–38. 10.1099/mic.0.2007/012856-0 18048907

[3] Pamukcu A Bush A Buchdahal R Effects of *P. aeruginosa* colonization on lung function and anthropomorphic variables in children with cystic fibrosis. Pediatr Pulmonol (1995) 19:10–5. 10.1002/ppul.1950190103 7675552

[4] Henry RL Mellis CM Petrovic L. Mucoid *Pseudomonas aeruginosa* is a marker of poor survival in cystic fibrosis. Pediatr Pulmonol (1992) 12:158–61. 10.1002/ppul.1950120306 1641272

[5] Balaguer A González de Dios J Home versus hospital intravenous antibiotic therapy for cystic fibrosis. Cochrane Database Syst Rev (2015) 12:CD001917 10.1002/14651858 PMC6481823 26671062

[6] Chmiel JF Aksamit TR Chotirmall SH Dasenbrook EC Elborn JS LiPuma JJ Antibiotic management of lung infections in cystic fibrosis. I. The microbiome, methicillin-resistant *Staphylococcus aureus*, Gram-negative bacteria, and multiple infections. Ann Am Thorac Soc (2014) 11:1120–9. 10.1513/AnnalsATS.201402-050AS 25102221 PMC5467101

[7] Langton Hewer SC Smyth AR Antibiotic strategies for eradicating *Pseudomonas aeruginosa* in people with cystic fibrosis. Cochrane Database Syst Rev (2014) 11:CD004197 10.1002/14651858 25383937

[8] Khan TZ Wagener JS Bost T Martinez J Accurso FJ Riches DW. Early pulmonary inflammation in infants with cystic fibrosis. Am J Respir Crit Care Med (1995) 151:1075–82. 10.1164/ajrccm.151.4.7697234 7697234

[9] Armstrong DS Grimwood K Carlin JB Carzino R Gutièrrez JP Hull J Lower airway inflammation in infants and young children with cystic fibrosis. Am J Respir Crit Care Med (1997) 156:1197–204. 10.1164/ajrccm.156.4.96-11058 9351622

[10] Pillarisetti N Williamson E Linnane B Skoric B Robertson CF Robinson P Infection, inflammation, and lung function decline in infants with cystic fibrosis. Am J Respir Crit Care Med (2011) 184:75–81. 10.1164/rccm.201011-1892OC 21493738

[11] Belessis Y Dixon B Hawkins G Pereira J Peat J MacDonald R Early cystic fibrosis lung disease detected by bronchoalveolar lavage and lung clearance index. Am J Respir Crit Care Med (2012) 185:862–73. 10.1164/rccm.201109-1631OC 22323305

[12] Wolter J Seeney S Bell S Bowler S Masel P McCornack J. Effect of long term treatment with azithromycin on disease parameters in cystic fibrosis: a randomised trial. Thorax (2002) 57:212–6. 10.1136/thorax.57.3.212 11867823 PMC1746273

[13] Equi A Balfour-Lynn IM Bush A Rosenthal M Long term azithromycin in children with cystic fibrosis: a randomised, placebocontrolled crossover trial. Lancet (2002) 360:978–84. 10.1016/S0140-6736(02)11081-6 12383667

[14] Saiman L Marshall BC Mayer-Hamblett N Burns JL Quittner AL Cibene DA Macrolide Study Group: azithromycin in patients with cystic fibrosis chronically infected with *Pseudomonas aeruginosa*: a randomised controlled trial. JAMA (2003) 290:1749–56. 10.1001/jama.290.13.1749 14519709

[15] Saiman L Anstead M Mayer-Hamblett N Lands LC Kloster M Hocevar-Trnka J Effect of azithromycin on pulmonary function in patients with cystic fibrosis uninfected with *Pseudomonas aeruginosa*: a randomized controlled trial. JAMA (2010) 303:1707–15. 10.1001/jama.2010.563 20442386

[16] Ratjen F Saiman L Mayer-Hamblett N Lands LC Kloster M Thompson V Effect of azithromycin on systemic markers of inflammation in patients with cystic fibrosis uninfected with *Pseudomonas aeruginosa*. Chest (2012) 142:1259–66. 10.1378/chest.12-0628 22595153 PMC3610595

[17] Gillis RI Iglewski BH. Azithromycin retards *Pseudomonas aeruginosa* biofilm formation. J Clin Microbiol (2004) 42:5842–5. 10.1128/JCM.42.12.5842-5845.2004 15583321 PMC535287

[18] Legssyer R Huaux F Lebacq J Delos M Marbaix E Lebecque P Azithromycin reduces spontaneous and induced inflammation in DeltaF508 cystic fibrosis mice. Respir Res (2006) 7:134. 10.1186/1465-9921-7-134 17064416 PMC1637104

[19] van Doorninck JH French PJ Verbeek E Peters RH Morreau H Bijman J A mouse model for the cystic fibrosis delta F508 mutation. EMBO J (1995) 14:4403–11. 7556083 10.1002/j.1460-2075.1995.tb00119.x PMC394531

[20] Nicklas W Baneux P Boot R Decelle T Deeny AA Fumanelli M Recommendations for the health monitoring of rodent and rabbit colonies in breeding and experimental units. Lab Anim (2002) 36:20–42. 10.1258/0023677021911740 11831737

[21] Bergamini G Di Silvestre D Mauri P Cigana C Bragonzi A De Palma A MudPIT analysis of released proteins in *Pseudomonas aeruginosa* laboratory and clinical strains in relation to pro-inflammatory effects. Integr Biol (Camb) (2012) 4:270–9. 10.1039/c2ib00127f 22298109

[22] Di Paolo A Barbara C Chella A Angeletti CA Del Tacca M. Pharmacokinetics of azithromycin in lung tissue, bronchial washing, and plasma in patients given multiple oral doses of 500 and 1000 mg daily. Pharmacol Res (2002) 46:545–50. 10.1016/S1043661802002384 12457629

[23] Lubamba B Huaux F Lebacq J Marbaix E Dhooghe B Panin N Immunomodulatory activity of vardenafil on induced lung inflammation in cystic fibrosis mice. J Cyst Fibros (2012) 11:266–73. 10.1016/j.jcf.2012.03.003 22503062

[24] Ducret A Van Oostveen I Eng JK Yates J III Aebersold R. High throughput protein characterization by automated reverse-phase chromatography/electrospray tandem mass spectrometry. Protein Sci (1998) 7:706–19. 10.1002/pro.5560070320 9541403 PMC2143958

[25] Carr S Aebersold R Baldwin M Burlingame A Clauser K Nesvizhskii A The need for guidelines in publication of peptide and protein identification data: Working Group on Publication Guidelines for Peptide and Protein Identification Data. Mol Cell Proteomics. (2004) 3:531–3. 10.1074/mcp.T400006-MCP200 15075378

[26] Mauri P Scarpa A Nascimbeni AC Benazzi L Parmagnani E Mafficini A Identification of proteins released by pancreatic cancer cells by multidimensional protein identification technology: a strategy for identification of novel cancer markers. FASEB J (2005) 19:1125–7. 10.1096/fj.04-3000fje 15985535

[27] Hilario M Kalousis A. Approaches to dimensionality reduction in proteomic biomarker studies. Brief Bioinform (2008) 9:102–18. 10.1093/bib/bbn005 18310106

[28] Jain AK Murty MN Flynn PJ Data clustering: a review. ACM Comput Surv (1999) 31:264–323. 10.1145/331499.331504

[29] Zhao Y Karypis G. Data clustering in life sciences. Mol Biotechnol (2005) 31:55–80. 10.1385/MB:31:1:055 16118415

[30] Tan HL Regamey N Brown S Bush A Lloyd CM Davies JC. The Th17 pathway in cystic fibrosis lung disease. Am J Respir Crit Care Med (2011) 184:252–8. 10.1164/rccm.201102-0236OC 21474644 PMC3381840

[31] Ding W Smulan LJ Hou NS Taubert S Watts JL Walker AK. s-Adenosylmethionine levels govern innate immunity through distinct methylation-dependent pathways. Cell Metab (2015) 22:633–45. 10.1016/j.cmet.2015.07.013 26321661 PMC4598287

[32] Hevia H Varela-Rey M Corrales FJ Berasain C Martínez-Chantar ML Latasa MU 5’-Methylthioadenosine modulates the inflammatory response to endotoxin in mice and in rat hepatocytes. Hepatology (2004) 39:1088–98. 10.1002/hep.20154 15057913

[33] Lopes RL Borges TJ Araújo JF Pinho NG Bergamin LS Battastini AM Extracellular mycobacterial DnaK polarizes macrophages to the M2-like phenotype. PLoS One (2014) 9:e113441. 10.1371/journal.pone.0113441 25419575 PMC4242626

[34] Meyer M Huaux F Gavilanes X van den Brûle S Lebecque P Lo Re S Azithromycin reduces exaggerated cytokine production by M1 alveolar macrophages in cystic fibrosis. Am J Respir Cell Mol Biol (2009) 41:590–602. 10.1165/rcmb.2008-0155OC 19244203

[35] Høiby N Diffuse panbronchiolitis and cystic fibrosis: east meets west. Thorax (1994) 49:531–2. 10.1136/thx.49.6.531 8016786 PMC474936

[36] Koyama H Geddes DM Erythromycin and diffuse panbronchiolitis. Thorax (1997) 52:915–8. 10.1136/thx.52.10.915 9404381 PMC1758435

[37] Jaffé A Bush A Anti-inflammatory effects of macrolides in lung disease. Pediatr Pulmonol (2011) 31:464–73. 10.1002/ppul.1076 11389580

[38] Retsema J Fu W. Macrolides: structures and microbial targets. Int J Antimicrob Agents (2001) 18(Suppl 1):S3–10. 10.1016/S0924-8579(01)00401-0 11574188

[39] Poehlsgaard J Douthwaite S. The bacterial ribosome as a target for antibiotics. Nat Rev Microbiol (2005) 3:870–81. 10.1038/nrmicro1265 16261170

[40] Tsai WC Hershenson MB Zhou Y Sajjan U. Azithromycin increases survival and reduces lung inflammation in cystic fibrosis mice. Inflamm Res (2009) 58:491–501. 10.1007/s00011-009-0015-9 19271151 PMC4164971

[41] Imperi F Leoni L Visca P Antivirulence activity of azithromycin in *Pseudomonas aeruginosa*. Front Microbiol (2014) 5:178 10.3389/fmicb.2014.00178 24795709 PMC4001013

[42] Pier G Grout M Zaidi TS Cystic fibrosis transmembrane conductance regulator is an epithelial cell receptor for clearance of *Pseudomonas aeruginosa* from the lung. Proc Natl Acad Sci U S A (1997) 94:12088–93. 10.1073/pnas.94.22.12088 9342367 PMC23711

[43] Schroeder TH Lee MM Yacono PW Cannon CL Gerçeker AA Golan DE CFTR is a pattern recognition molecule that extracts *Pseudomonas aeruginosa* LPS from the outer membrane into epithelial cells and activates NF-kB translocation. Proc Natl Acad Sci U S A (2002) 99:6907–12. 10.1073/pnas.092160899 11997458 PMC124502

[44] Pier GB CFTR mutations and host susceptibility to *Pseudomonas aeruginosa* lung function. Curr Opin Microbiol (2002) 5:81–6. 10.1016/S1369-5274(02)00290-4 11834374

[45] Kelly NM MacDonald MH Martin N Nicas T Hancock RE. Comparison of the outer membrane protein and lipopolysaccharide profiles of mucoid and nonmucoid *Pseudomonas aeruginosa*. J Clin Microbiol (1990) 28:2017–21. 2121789 10.1128/jcm.28.9.2017-2021.1990 PMC268096

[46] Jekel PA Weijer WJ Beintema JJ. Use of endoproteinase Lys-C from *Lysobacter* enzymogenes in protein sequence analysis. Anal Biochem (1983) 134:347–54. 10.1016/0003-2697(83)90308-1 6359954

[47] Létoffé S Redeker V Wandersman C. Isolation and characterization of an extracellular haem-binding protein from *Pseudomonas aeruginosa* that shares function and sequence similarities with the *Serratia marcescens* HasA haemophore. Mol Microbiol (1998) 28:1223–34. 10.1046/j.1365-2958.1998.00885.x 9680211

[48] Marvig RL Damkiaer S Khademi SM Markussen TM Molin S Jelsbak L. Within-host evolution of *Pseudomonas aeruginosa* reveals adaptation toward iron acquisition from hemoglobin. mBio (2014) 5:e966–914. 10.1128/mBio.00966-14 24803516 PMC4010824

[49] Motta A Schmitz C Rodrigues L Ribeiro F Teixeira C Detanico T Mycobacterium tuberculosis heat-shock protein 70 impairs maturation of dendritic cells from bone marrow precursors, induces interleukin-10 production and inhibits T-cell proliferation in vitro. Immunology (2007) 121:462–72. 10.1111/j.1365-2567.2007.02564.x 17346283 PMC2265970

[50] Stocki P Wang XN Dickinson AM. Inducible heat shock protein 70 reduces T cell responses and stimulatory capacity of monocyte-derived dendritic cells. J Biol Chem (2012) 287:12387–94. 10.1074/jbc.M111.307579 22334699 PMC3320988

[51] Spiering R van der Zee R Wagenaar J van Eden W Broere F. Mycobacterial and mouse HSP70 have immuno-modulatory effects on dendritic cells. Cell Stress Chaperones (2013) 18:439–46. 10.1007/s12192-012-0397-4 23269491 PMC3682017

[52] Chu J Qian J Zhuang Y Zhang S Li Y. Progress in the research of S-adenosyl-l-methionine production. Appl Microbiol Biotechnol (2013) 97:41–9. 10.1007/s00253-012-4536-8 23135229

[53] Hueck CJ. Type III protein secretion systems in bacterial pathogens of animals and plants. Microbiol Mol Biol Rev (1998) 62:379–433. 9618447 10.1128/mmbr.62.2.379-433.1998 PMC98920

[54] Barbieri JT Sun J. *Pseudomonas aeruginosa* ExoS and ExoT. Rev Physiol Biochem Pharmacol (2004) 152:79–92. 10.1007/s10254-004-0031-7 15375697

[55] Sundin C Thelaus J Bröms JE Forsberg A Polarisation of type III translocation by *Pseudomonas aeruginosa* requires PcrG, PcrV and PopN. Microb Pathog (2004) 37:313–22. 10.1016/j.micpath.2004.10.005 15619427

[56] Engel J Balachandran P. Role of *Pseudomonas aeruginosa* type III effectors in disease. Curr Opin Microbiol (2009) 12:61–6. 10.1016/j.mib.2008.12.007 19168385

[57] Woods DE Hwang WS Shahrabadi MS Que JU. Alteration of pulmonary structure by *Pseudomonas aeruginosa* exoenzyme S. J Med Microbiol (1988) 26:133–41. 10.1099/00222615-26-2-133 3133480

[58] Woods DE Schaffer MS Rabin HR Campbell GD Sokol PA. Phenotypic comparison of *Pseudomonas aeruginosa* strains isolated from a variety of clinical sites. J Clin Microbiol (1986) 24:260–4. 3018037 10.1128/jcm.24.2.260-264.1986 PMC268885

[59] Sun Y Karmakar M Taylor PR Rietsch A Pearlman E. ExoS and ExoT ADP ribosyltransferase activities mediate *Pseudomonas aeruginosa* keratitis by promoting neutrophil apoptosis and bacterial survival. J Immunol (2012) 188:1884–95. 10.4049/jimmunol.1102148 22250085 PMC3273577

[60] Verove J Bernarde C Bohn YS Boulay F Rabiet MJ Attree I Injection of *Pseudomonas aeruginosa* Exo toxins into host cells can be modulated by host factors at the level of translocon assembly and/or activity. PLoS One (2012) 7:e30488. 10.1371/journal.pone.0030488 22299042 PMC3267729

[61] Stellari F Bergamini G Sandri A Donofrio G Sorio C Ruscitti F In vivo imaging of the lung inflammatory response to *Pseudomonas aeruginosa* and its modulation by azithromycin. J Transl Med (2015) 13:251. 10.1186/s12967-015-0615-9 26239109 PMC4522964

